# Soil respiration and controls in warmer winter: A snow manipulation study in postfire and undisturbed black pine forests

**DOI:** 10.1002/ece3.11075

**Published:** 2024-03-06

**Authors:** Renato S. Pacaldo, Mirac Aydin, Randell Keith Amarille

**Affiliations:** ^1^ Faculty of Forestry Kastamonu University Kastamonu Turkey; ^2^ College of Forestry and Environmental Studies Mindanao State University Marawi City Philippines

**Keywords:** air temperature, climate change, freeze–thaw, soil moisture, soil temperature, wildfire

## Abstract

Climate change impacts drive warmer winters, reduced snowfall, and forest fires. In 2020, a wildfire scorched about 1508 hectares of black pine (*Pinus nigra* Arnold) forests in Türkiye. Whether the combined effects of lack of snow and forest fires significantly alter winter soil respiration (R_s_) and soil temperature remains poorly understood. A field experiment was conducted in the postfire and undisturbed black pine forests during the winter to quantify R_s_ rates as affected by lack of snow and forest fire. We applied four treatments: snow‐exclusion postfire (SEPF), snow postfire (SPF), snow‐exclusion‐undisturbed forest (SEUF), and snow undisturbed forest (SUF). The SEPF exhibited the significantly lowest mean R_s_ rates (0.71 μmol m^−2^ s^−1^) compared to the SPF (1.02 μmol m^−2^ s^−1^), SEUF (1.44 μmol m^−2^ s^−1^), and SUF (1.48 μmol m^−2^ s^−1^). The R_s_ also showed significant variations with time (*p* < .0001). However, treatments and time revealed no statistically significant interaction effects (*p* = .6801). Total winter R_s_ (January–March) ranged from 4.47 to 4.59 Mt CO_2_ ha^−1^ in the undisturbed forest and 2.20 to 3.16 Mt CO_2_ ha^−2^ in the postfire site. The R_s_ showed a significantly positive relationship (*p* < .0001) with the soil (0.59) and air (0.46) temperatures and a significantly negative relationship (*p* = .0017) with the soil moisture (−0.20) at the 5 cm depth. In contrast, the R_s_ indicated a negative but not statistically significant relationship (*p* = .0932) with the soil moisture (−0.16) at the 10 cm soil depth. The combined effects of lack of snow and forest fire significantly decreased R_s_, thus conserving the soil's organic carbon stocks and reducing the CO_2_ contribution to the atmosphere. In contrast, a warmer winter significantly increased R_s_ rates in the undisturbed forest, suggesting an acceleration of soil organic carbon losses and providing positive feedback to climate change.

## Graphical abstract

This study addresses a critical question of whether forest and lack of snow would significantly alter soil respiration (R_s_) rates in postfire and undisturbed black pine forests. Results revealed that the combined effects of lack of snow and forest fire caused a significant decrease in R_s_ rates. In contrast, a warmer winter significantly increased R_s_ rates in the undisturbed forest, suggesting that a warmer winter could potentially accelerate soil organic carbon losses in naturally growing undisturbed forest ecosystems, thus providing positive feedbacks to climate change.

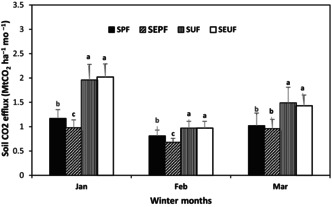

## 1 INTRODUCTION

Increasing temperatures, decreasing snow cover, increasing hot summer days, warmer winters, and other natural disasters such as forest fires, floods, super typhoons, and hurricanes have been linked to global climate change impacts (Haei & Laudon, 2015). Some authors predicted that the global temperature will increase between 1.4 and 5.8°C by 2100 (Houghton et al., 2001; IPCC, 2001; Stocker, 2014) and is expected to modify natural processes, alter precipitation patterns, and reduce snowfall (Croce et al., 2018; Zhang, 2005). With this projected scenario, the Intergovernmental Panel for Climate Change (IPCC) strengthened the climate change response of the global community by pursuing efforts within the framework of sustainable development goals that would limit the global temperature increase to 1.5°C above the pre‐industrial level (Masson‐Delmotte et al., 2022). The IPCC has been promoting carbon sequestration in terrestrial vegetation as one of the measures to mitigate the impacts of climate change. Green plants absorb atmospheric carbon dioxide (CO_2_) through photosynthesis and store it in biomass components (IPCC, 2001; Justine et al., 2015). However, the carbon sequestration capacity of terrestrial vegetation could be altered by warmer climates, lack of snow, and forest fires (Campbell et al., 2005; Croce et al., 2018), which could modify net primary productivity (Yang et al., 2018) and soil microorganism activities (Yang et al., 2022).

In Türkiye, climate change impacts have been driving an increased surface temperature (Demircan et al., 2017; Gorguner et al., 2019), decreased precipitation (Turkes et al., 2020), particularly in southern half of the country with projected reduction by 37% in the Mediterranean basins, 70% in the Konya basin, and up to 10% in the Euphrates and Tigris basins by the mid‐21st century (Şen et al., 2014), and extreme but fewer drought events in the Ankara Province (Danandeh Mehr et al., 2020). The warmer summer temperature in Türkiye has also increased forest fires across the country, destroying 222,384 hectares of forestland from 2010 to 2021 (Memisoglu Baykal, 2023). Some authors projected that these climate change‐related impacts would continue in the next few decades due to the ongoing temperature anomalies in the country, with predicted temperature increases between 2 and 6°C (Demircan et al., 2017; Önol et al., 2014). The increasing surface temperature has led to high evaporation rates in the Black Sea that caused intense flooding across the country, particularly in areas bordering the sea (Nuri Balov & Altunkaynak, 2019). Furthermore, projections of future climate trajectory in the country also estimated lesser amounts of snowfall in the interior part of the Black Sea region because of the orographic effects caused by natural barriers consisting of high mountains lying parallel to the shoreline of the Black Sea (Aksu et al., 2022; Nuri Balov & Altunkaynak, 2019).

The changes in precipitation, coupled with increased air temperature, are expected to drive long dry days (Nuri Balov & Altunkaynak, 2019) and a warmer winter and soil that would drive frequent freeze–thaw cycles, hence affecting soil respiration (R_s_) rates, the main pathway in the carbon cycle in which CO_2_ emissions from soil‐borne autotrophic and heterotrophic organisms escape into the atmosphere. Although R_s_ rates could vary due to differences in plants' adaptability and productivity rates (Li et al., 2013; Pacaldo & Aydin, 2023) and availability of dissolved organic carbon and microbial biomass carbon (Liu et al., 2019), many studies demonstrated that the soil temperature strongly influences R_s_ rates. Some authors observed that an increase in soil temperature by 2°C results in the rise of R_s_ rates by about 12%–21% (Wang et al., 2014). A field warming experiment also demonstrated that warming significantly enhanced autotrophic and total respiration rates but reduced heterotrophic respiration (Chen et al., 2016).

A warmer climate in semi‐arid and arid regions could increase or decrease precipitation rates. An increased precipitation rate expands the area of land carbon sinks (Poulter et al., 2014) and accelerates R_s_ rates due to the favorable response of heterotrophic organisms to abundant soil moisture contents (Du et al., 2018). By contrast, a decreased precipitation due to a warmer climate abates R_s_ rates due to reduced autotrophic respiration (Du et al., 2023) and reduces snow cover, particularly in areas fronting natural barriers in which orographic effects and local topography affect snow patterns (Campbell et al., 2005; Croce et al., 2018).

A warmer climate also triggers frequent forest fires, affecting R_s_ rates and soil C budgets. Forest fires result in the destruction of autotrophic and heterotrophic organisms and significantly remove organic matter and soil carbon stocks, which in turn leads to the loss of nutrients through volatilization, increased leaching and erosion, and alteration of quantity and composition of microbial and other soil organisms (Certini, 2005). Depending on the fire intensity, soil heating brings about severe modifications in the physical and chemical properties of the soil and other residual matter and affects microbial activities and other soil‐borne organisms (Ahlgren, 1974; Masyagina et al., 2016), particularly in the top 10 cm depth (Cowan et al., 2016). However, reported observations on the effects of fire on R_s_ rates are not consistent, with some authors reporting an increase in R_s_ due to enhanced heterotrophic respiration rates (Hu et al., 2021; Wang, Chen, et al., 2021; Wang, Yao, et al., 2021) while others reporting a decrease due to suppressed microbial activities and reduced microbial biomass (Chen et al., 2019; Hu et al., 2023). Kong et al. (2019) reported no R_s_ changes regardless of fire intensity. Some authors attributed the increased R_s_ rates to the release of high amounts of macronutrients, except nitrogen, and the ash deposit raises the soil pH, hence creating a soil environment favorable for the growth of heterotrophic organisms (Bárcenas‐Moreno et al., 2011; Ernfors et al., 2010; Moilanen et al., 2012) and promoting early recruitment and recolonization of pioneering plants (Dzwonko et al., 2015; Silvan & Hytönen, 2016).

In Türkiye, forest fires destroyed large tracks of forestlands, mainly the naturally growing black pine (*Pinus nigra* Arnold) forests. In 2020, a wildfire destroyed about 1508 hectares of natural black pine forests in the Taşköprü Forest Directorate, Kastamonu District, Türkiye. The black pine is one of the most abundant and valuable timber species in Türkiye, with an estimated area of about 4.2 million hectares across the Anatolia and Black Sea regions and in the northern part of the Taurus mountains. The Kastamonu region has the largest area of black pine forest, with an estimated area of 1.26 million hectares or about 66% of black pine forests in the country, producing about 200 million cubic meters or 13% of the total wood production (Sakici et al., 2018).

Given that a warmer climate is causing lesser amounts of snow deposits, driving frequent freeze and thaw cycles, altering precipitation patterns, and bringing about frequent forest fires, particularly in Türkiye, a critical question is whether these climate change‐related impacts result in a significant alteration of R_s_ in standing undisturbed forest ecosystems and forest fire‐disturbed areas. Despite the growing interest in R_s_ studies and recognition of the importance of R_s_ in accounting for the global C budget, this component of the C cycle during winter is significantly understudied (Graham & Risk, 2018). Although several studies documented R_s_ rates during winter in different types of forest ecosystems in temperate regions (e.g., Brooks et al., 1996; Coxson & Parkinson, 1987; Liptzin et al., 2009; Nielsen et al., 2001; Pacaldo, 2012; Sommerfeld et al., 1993; Taylor & Jones, 1990), there have been no detailed studies or experimental manipulations focused on the effects of lack of snow or milder winter freeze on R_s_ rates and its controlling factors in forest fire sites and undisturbed black pine forests. This shortcoming precluded understanding of the magnitude of R_s_ rates in seasonally snow‐covered forests in semi‐arid regions, particularly in Türkiye, in which climate change impacts have been strongly affecting snow deposits during winter and the severe problem of forest fires during summer due to dry and warm temperature.

To reduce the uncertainty above, we present here the results of the winter season R_s_ measurements in one of the high‐elevation postfire sites and undisturbed natural forests in Taşköprü, Kastamonu District, Türkiye. We aimed to quantify the R_s_ rates, soil temperature, air temperature, and soil moisture during winter in the snow‐free (snow‐sheltered) and snow‐exposed (control) sites in postfire and undisturbed natural black pine forests. We posited that the lack of snow, as may occur due to climate change impacts, will cause significant differences in R_s_ rates in postfire and undisturbed natural forests because of the differences in soil temperatures, with warmer soils in undisturbed forests and colder soils in postfire areas. Thick layers of forest litter in the undisturbed forest protect and keep the soil surface warm. At the same time, a lack of snow cover exposes the soil surface to direct contact with air temperatures. Cold air freezes the soil surface, sealing soil pores, preventing soil CO_2_ diffusion, and inhibiting soil heterotrophic respirations. Understanding the combined effects of lack of snow and forest fires on R_s_ rates is essential for projecting the future trajectory of C budgets and climate‐R_s_ feedbacks in forest ecosystems and postfire sites. To our knowledge, this study was the first investigation on R_s_ rates in the black pine forest during winter, both in the undisturbed and postfire sites.

## 2 METHODS

### 2.1 Study location

The research site is located at a recently burned black pine forest (*Pinus nigra* Arnold) within the Taşköprü Forest Directorate, Kastamonu District, Türkiye, geographically situated between 41°19′02″ N latitude and 34°08′48″ E longitude, with an elevation of 1054 m above sea level (masl) and slopes ranging from 0% to 45%. In 2020, a wildfire scorched the research site, destroying about 1508 hectares of naturally growing black pine forests. This study's postfire area is a burned forest site that eventually becomes covered with grasses and newly growing wildlings. The soil is covered with ashes, charred wood debris, and decomposing organic matter. In contrast, the undisturbed natural forest refers to the standing forest bordering the postfire area fully covered by trees of mixed ages with layers of organic matter (OM) on the soil surface. These sites were chosen as our study sites because they receive much snowfall that stays on the ground throughout winter due to high mountain elevations and freezing temperatures.

### 2.2 Site characteristics

Based on the meteorological data, Kastamonu–Taşköprü's climate is a marine west coast with warmer summer (Cfb) and air temperatures between −24 and 21°C during the winter months (January–March). Generally, the Kastamonu–Taşköprü District usually has a mean annual temperature and precipitation of 9.7°C and 413.2 mm, respectively (Cigdem et al., 2023). In this study, the winter was unusually warmer than previous winter seasons, with measured air temperatures ranging from −2.9 to 16.7°C and soil temperatures ranging from −0.2 to 9.1°C for the 5 cm soil depth and −2.5 to 12.9°C for the 10 cm soil depth. The snow depth was largely variable across the landscape. During the measurement period, the site received less snow than in previous years due to dry and warmer weather conditions.

Areas encompassed in the research site were similar regarding management history, climatic conditions, and vegetation consisting of pure black pines in multi‐age classes. Based on our field measurements, the stand density of the forest ranged from 425 to 3375 trees per hectare with diameter breast height (dbh) ranging from 1 to 136 cm. Weeds, vines, grasses, and wildlings have invaded the postfire site. Depending on the position in the landscape, the effective rooting depth ranged from 30 to 40 cm depth on the upslope and 45 to 60 cm in the mid‐slope (20%–45% slopes).

In each sampling plot, composite soil samples, taken diagonally across the sampling plot using a cylindrical bulk density corer (5 cm dia × 5 cm ht), were also collected at two layer depths (0–15 and 16–30 cm) of the mineral soil. The soil texture was determined using a hydrometer method for particulate size analysis. The soil bulk density was calculated by dividing the dry weight of soil samples (corrected for stones) by the volume of soil corer (inside diameter). The soil organic matter content in the mineral soil was determined by the loss‐of‐ignition (LOI) method. The SOM was converted into soil organic carbon (SOC) by dividing the SOM by 1.724, based on the assumption that the SOM contains 58% carbon (Nelson, 1983; Post et al., 1982).

Forest litter samples on the soil surface, ranging in depths from 10 to 20 cm in undisturbed forest and 0 to 2 cm in postfire, were also collected using a litter sampler (25 cm × 25 cm) to estimate the quantity of accumulated forest litter on the soil surface in the undisturbed forests and postfire areas. Samples of forest litter were oven‐dried at 65°C to constant weight and then scaled up the dry weights to metric tons per hectare (Mt OM ha^−1^). The SOM values were converted to SOC using a conversion factor of 0.51, based on the report that the mean C contents of the aboveground biomass components of black pines are 51 percent (Sakici et al., 2018; Tolunay, 2019). The results of the analyses are summarized below (Table 1).

**TABLE 1:** Physical and chemical properties of soils in the undisturbed forest and postfire (*n* = 4; mean ± SE).

	Soil depth (cm)	Bulk density (g cm^−3^)	SOM (Mt ha^−1^)	SOC (Mt ha^−1^)	pH	EC	Texture
Undisturbed forest	Surface^a^		127.7 (26.3)	65.1 (13.4)			
	0–15	1.1 (0.1)	172.9 (18.8)	100.3 (10.9)	4.4 (0.2)	106.4 (5.7)	SL
	16–30	1.2 (0.2)	84.6 (12.8)	49.1 (7.4)	4.5 (0.1)	184.6 (39.4)	SCL
Postfire	Surface^b^		26.0 (10.7)	13.3 (5.5)			
	0–15	0.9 (0.1)	176.9 (12.4)	102.6 (7.2)	4.4 (0.1)	143.7 (12.3)	SCL
	16–30	1.2 (0.1)	82.2 (7.9)	47.7 (4.6)	5.0 (0.1)	185.9 (23.1)	SC

Abbreviations: SC, sandy clay; SCL, sandy clay loam; SL, sandy loam.

^a^ 10–15 cm thick of forest litter.

^b^ 1–2 cm thick of grass litter.

### 2.3 Experimental design and treatments

We established the experiment in a split‐plot design with the research site as a whole plot and four subplots as a split plot. Each subplot had square dimensions of 2 m × 2 m with 16 subplots to accommodate all treatments and four replications (4 × 4). The treatments included snow postfire (SPF), snow‐exclusion postfire (SEPF), snow undisturbed forest (SUF), and snow‐exclusion‐undisturbed forest (SEUF). We used the SPF and SUF as control plots for the postfire and undisturbed forest sites. The control plots were left exposed to natural weather conditions and snowfall during the winter season. In contrast, the SEPF and SEUF were the snow‐exclusion plots sheltered by snow‐exclusion chambers. Each chamber was constructed using a wooden A‐frame (1 m length × 1 m width × 1 m height) and was fully covered by transparent plastic sheets, except the area within the bounds of the A‐frames, which serve as air vents to allow unrestricted airflow and interactions between inside and outside environments of the chamber.

In each subplot, two cylindrical polyvinyl chloride (PVC) soil collars (5 cm diameter × 5 cm height) with sharpened edges at the lower end were inserted into the soil at a depth of about 2 cm, leaving a collar headspace of about 3 cm above the soil surface. We installed a total of 32 soil collars in the field a week before the start of measurements. Sixteen soil collars were exposed to snowfall, while the snow‐exclusion chambers sheltered the other half. The shelters were constantly maintained and repaired whenever they sustained damages due to strong winds.

### 2.4 Soil respiration measurement

We measured R_s_ over a discrete PVC soil collar in which the headspace CO_2_ emissions were analyzed using an automated soil respiration machinery (LI‐8100A), consisting of infrared gas (IRGA) analyzer unit (LI‐8100), survey chamber (LI‐8100‐103), soil temperature probe (6000‐09TC Omega), and EC‐5 soil moisture sensor (Decagon Devices, Pullman, WA, USA) (LI‐COR Biosciences). During winter R_s_ measurements, we gently removed the snow on the top of soil collars in the unsheltered plots (control) to allow the mounting of the survey chamber and then covered immediately with some snowpack to keep the soil collar under snow cover throughout the measurement period. We measured the soil temperature and volumetric soil moisture content (VMC) at two soil depths (50 and 15 cm) with R_s_. The R_s_ measurement for each soil collar lasted for 240 s, consisting of 30 s of equilibration/deadband (i.e., the length of time when a chamber closes completely and mixes with air before measurement begins), a 150‐s observation length, and 60 s of purge time (i.e., time in which air continues to flow through the chamber as it opens following the observation length) (Pacaldo et al., 2014). Measured data are available in Appendix S1.

Before treatments were applied, baseline soil respiration (R_s_) measurements were conducted in the late spring to early summer to determine differences in R_s_ rates in sampling plots. Results revealed that R_s_ rates ranged from 1.59 to 2.33 μmol m^−2^ s^−1^ for the undisturbed forest and 1.53 to 2.64 μmol m^−2^ s^−1^ in the postfire forest area, but statistical analyses detected no significant differences (*p* = .9959) among the different sites.

During the winter period, the R_s_ measurements were conducted twice a month following every snow event, based on the weather forecast, to ensure a thick snow cover in snow‐exposed treatment plots, our primary treatment. Although the alternating snow freezing and thawing events occurred frequently in lowland areas because of alternating cold and warm weather conditions, the research site remained under snow cover throughout the study because of persistent freezing air temperature owing to the high mountain elevations. Our analyses also revealed that the variability of R_s_ rates ranged from 50% to 75% for January and 27% to 45% for February and March. Due to the homogeneity of temperature, persistency of snow cover, and lower variability of R_s_, except January, we assumed that twice‐a‐month measurement would provide reasonable average rates to represent the R_s_ rates during this period. Furthermore, the research site was inaccessible by vehicles without fresh snow to cover the road pavement due to hardened snow (black ice) on the road surface during periods of lack of snowfall.

R_s_ rates (μmol s^−1^ m^−2^) were scaled to metric tons CO_2_ per hectare by converting micromole to mole and then into grams CO_2_ (1 mole = 44 g), expressed in metric tons per month per hectare in (1 month = 30 days; 1 ha = 10,000 m^2^; 1 short ton = 0.9072 metric ton). The soil organic matter equivalents (SOM_eqv_) were calculated by dividing the soil CO_2_ (Mt CO_2_ mo^−1^ ha^−1^) by 1.87 (i.e., (44 g CO_2_/12 g C) × 51% C in SOM). SOC equivalents were computed by multiplying SOM by 0.58 (proportion of C in SOM) (Nelson, 1983; Post et al., 1982). The cumulative CO_2_, SOM, and SOC (Mt ha^−1^) values were determined as the daily cumulative R_s_ for January, February, and March.

### 2.5 Statistical analysis

We analyzed the effects of snow on soil respiration rates with a general linear model. We considered time a second qualitative factor to test whether R_s_ rates vary among different measurement times, treatments as fixed effects, and plots as random effects. The relationships among soil respiration, soil temperature, air temperature, and soil moisture were analyzed using regression and correlation analyses based on collected data throughout the study. We used the *r* ^2^ values, Mallows' C_p_ statistics, Akaike's information criterion (AIC), and mean standard error (MSE) to select the number of independent variables in the multiple regression model. We also performed a univariate analysis and Levene's test to determine the normality of data distribution and homogeneity of variance, respectively.

We tested the hypotheses of no significant differences in R_s_ rates, soil temperatures (5 and 10 cm depths), moisture contents, and their interaction effects in all treatments using an analysis of variance (ANOVA) with *p* < .05 considered as a significant value. A multiple‐wise comparison, using Tukey's test, was used to separate significant differences among treatment means. All statistical analyses were performed using a SAS Statistical Package (SAS 9.1 SAS Institute).

## 3 RESULTS

### 3.1 Soil respiration rates

The winter R_s_ showed significant differences among treatments (*p* < .0001) and varied across times of measurement (*p* < .0001). However, treatment and time exhibited no significant interaction effects (*p* = .6801), suggesting a consistency of relationships between the times of measurement and treatments (Table 2).

**TABLE 2:** Results of the analysis of variance (ANOVA) showing main treatment effects and interaction effects between R_s_ and time.

Source of variation	df	MS	*F*‐value	*p*‐value
Treatments	3	3.19	11.05	<.0001
Measurement period (time)	5	2.45	8.47	<.0001
Treatment × time	18	0.22	0.79	0.6801

**FIGURE 1:**
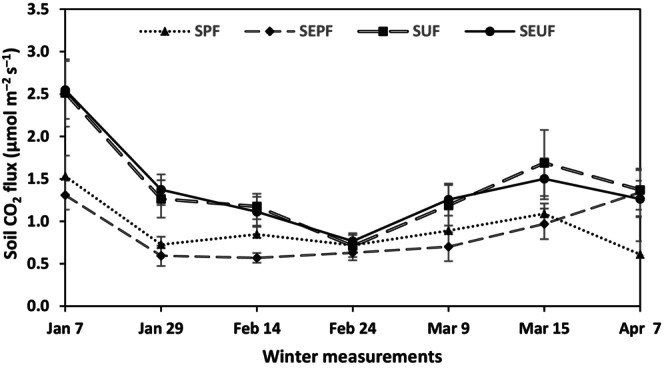
Temporal patterns of soil respiration rates (soil CO_2_ efflux) in the snow postfire (SPF) (control), snow‐exclusion postfire (SEPF), snow undisturbed forest (SUF) (control), and snow‐exclusion‐undisturbed forest (SEUF). Values are mean with standard error (mean ± SE) (*n* = 16).

**FIGURE 2:**
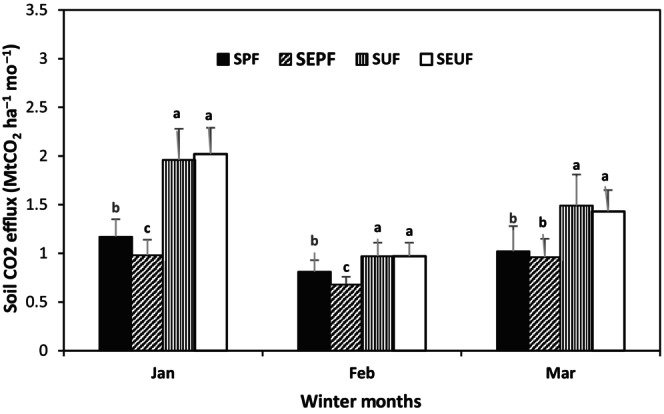
Winter season (January–March 2023) soil respiration rates (MtCO_2_ ha^−1^) in the postfire forest area exposed to snow, the postfire area with snow‐exclusion roofing (SEPF), undisturbed natural forest exposed to snow (SUF), and undisturbed natural forest with snow‐exclusion roofing (SEUF). Values are means (with standard error) of two measurements per month with four plots (replications) per treatment (*n* = 8 per month). The same letter indicates no significant differences among treatments at *p* < .05.

The respective monthly mean R_s_ values for January, February, and March were 1.13, 0.78, and 0.99 μmol s^−1^ m^−2^ for snow postfire (SPF); 0.95, 0.60, and 0.84 μmol s^−1^ m^−2^ for snow‐exclusion postfire (SEPF); 1.89, 0.94, and 1.44 μmol s^−1^ m^−2^ for snow undisturbed forest (SUF); and 1.96, 0.94, and 1.38 μmol s^−1^ m^−2^ for snow‐exclusion‐undisturbed forest (SEUF) (Figure 1). Although the R_s_ values in all treatments were generally low, the postfire site exhibited the lowest R_s_ rates from January until the middle of winter (February) compared with the undisturbed forests. R_s_ rates in the SUF and SEUF treatments showed no significant differences on a monthly average. Throughout the study, they exhibited significantly higher R_s_ than SPF and SEPF (postfire). In contrast, the R_s_ of SPF and SEPF showed significant differences in January and February but not in March (Figure 2).

The mean Rs, calculated as the average effluxes from January to March, revealed the highest R_s_ in SUF (1.48 μmol m^−2^ s^−1^), followed by SEUF (1.44 μmol m^−2^ s^−1^) and SPF (1.02 μmol m^−2^ s^−1^), and the lowest R_s_ in SEPF (0.71 μmol m^−2^ s^−1^). The SUF and SEUF exhibited no significant differences in the mean R_s_ but were significantly higher than the SPF and SEPF. The cumulative R_s_ and their equivalents SOM and SOC, calculated as the sum of daily cumulative R_s_ rates from January to March, showed that the undisturbed forests (4.59 Mt CO_2_ ha^−1^ for SEUF and 4.47 Mt CO_2_ ha^−1^ for the SUF) had higher R_s_ rates (44%) than the SEPF (2.20 Mt CO_2_ ha^−1^) and 32% than the SPF (3.16 Mt CO_2_ ha^−1^) (Table 3).

**TABLE 3:** Mean soil respiration (R_s_) and cumulative R_s_ rates (MtCO_2_ ha^−2^) with their corresponding soil organic matter and soil organic carbon equivalents, calculated as the sum of daily cumulative R_s_ rates from January to March.

Treatments	Mean R_s_ (μmol m^−2^ s^−1^)	Cumulative R_s_ (MtCO_2_ ha^−1^)	SOM equivalent (Mt SOM_eqv_ ha^−1^)	SOC equivalent (Mt SOC_eqv_ ha^−1^)
SPF	1.02^b^	3.16 (0.15)	1.69 (0.26)	0.98 (0.08)
SEPF	0.71^c^	2.20 (0.13)	1.18 (0.21)	0.67 (0.07)
SUF	1.48^a^	4.59 (0.25)	2.46 (0.13)	1.42 (0.08)
SEUF	1.44^a^	4.47 (0.20)	2.39 (0.11)	1.39 (0.06)

*Note*: Values inside the parenthesis represent the cumulative mean, standard error (±SE) (*n* = 24). Mean R_s_ with same letter not significantly different at 95% probability level, based on LSD test.

Abbreviations: SEPF, snow‐exclusion postfire (with snow shelter); SEUF, snow‐exclusion‐undisturbed forest (with snow shelter); SPF, snow postfire (control); SUF, snow undisturbed forest (control).

### 3.2 Soil temperature, air temperature, and soil moisture

Mean soil temperatures for the entire winter season revealed no significant differences among treatments, with average values ranging from 3.15 to 4.36°C in the 5 cm depth (*p* = .3210) and 3.72 to 4.54°C in the 10 cm depth (*p* = 0. 3472). Similarly, the mean air temperature, ranging from 4.18 to 5.31°C, showed no significant differences (*p* = .8162). The soil moisture at the 10 cm depth also showed no significant differences (*p* = .0932). Conversely, the 5 cm depth of the SEUF indicated a significantly lower moisture content (*p* = .0017) compared with the other treatments (Table 4).

**TABLE 4:** Mean soil temperature, moisture content at 5 and 10 cm depths, and air temperature during winter (January–March).

Treatments	Mean soil temp. at 5 cm depth (°C)	Mean soil temp. at 10 cm depth (°C)	Mean air temp. (°C)	Mean vol. soil moisture at 5 cm depth (%)	Mean vol. soil moisture at 10 cm depth (%)
SPF	3.53^a^	3.76^a^	4.18^a^	51.38^a^	46.91^a^
SEPF	3.15^a^	3.72^a^	4.36^a^	45.85^a^	43.30^a^
SUF	4.36^a^	4.54^a^	5.31^a^	43.92^a^	42.66^a^
SEUF	3.99^a^	4.13^a^	5.31^a^	37.72^b^	37.99^a^

*Note*: Mean values with the same letters indicate there have been no significant differences at a 95% probability level, based on the LSD test.

Abbreviations: SEPF, snow‐exclusion postfire forest (with snow shelter); SEUF, snow‐exclusion‐undisturbed forest (with snow shelter); SPF, snow postfire forest (control); SUF, snow undisturbed forest (control).

**FIGURE 3:**
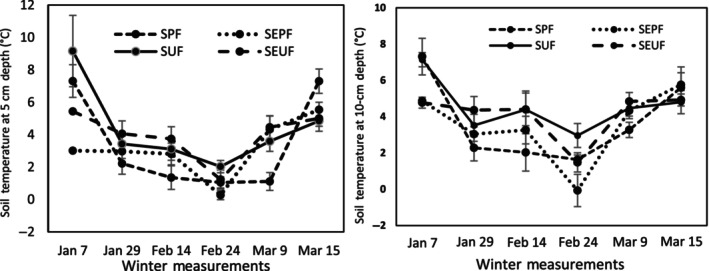
Soil temperature trends in the 5 and 15 cm depths from January to March in all four treatments. Values are the mean (with standard error) of four treatment plots per measurement. Bars with the same letters are not significantly at *p* < .05.

**FIGURE 4:**
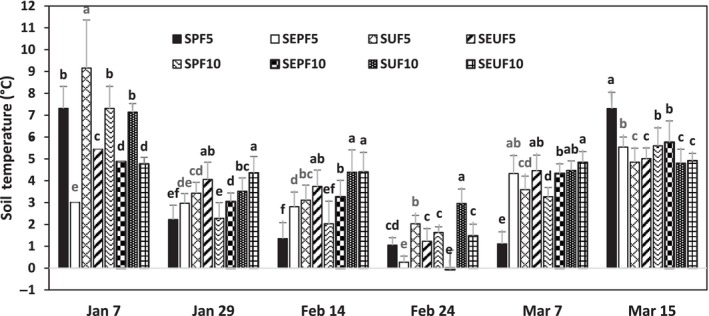
Comparison of monthly soil temperature in all depths and treatments. Values are the mean values of soil temperature (with standard error of the mean). Bars with the same letters are not significantly at *p* < .05. SPF5 means snow postfire at 5 cm depth; SPF10 snow postfire at 10 cm depth; SEPF5 snow exclusion postfire at 5 cm depth; SEPF10 snow exclusion postfire at 10 cm depth; SUF5 snow undisturbed forest at 5 cm depth; SUF10 snow undisturbed forest at 10 cm depth; SEUF5 snow exclusion undisturbed forest at 5 cm depth; and SEUF10 snow exclusion undisturbed forest at 10 cm depth.

However, it is apparent that from the outset of the study, the postfire sites already exhibited significantly lower temperatures than the undisturbed forests, both in the 5 and 15 cm depths. The soil temperatures in all treatments steadily decreased to nearly 0°C in the middle of the winter season onwards, except for the 15 cm depth of the snow‐free plots in which the soil temperature dropped to below freezing temperature. In contrast, the soil temperatures in the undisturbed forest remained above 2°C, both in the 5 and 15 cm depths, except in the middle of winter, wherein the temperature settled slightly above 1°C (Figures 3 and 4).

### 3.3 Relationships between soil respiration, temperature, and soil moisture

**FIGURE 5:**
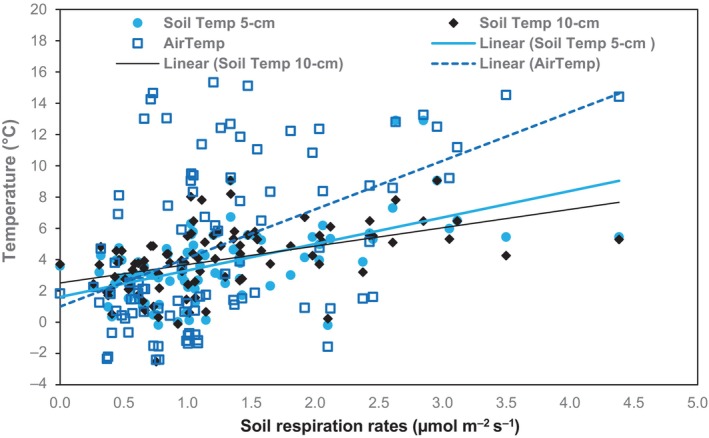
Soil respiration rates (μmol m^−2^ s^−1^) as a function of soil temperatures (°C) at 5 and 10 cm depths and the air temperature (°C). The correlations show moderately strongly positive relationships of R_s_ rates with soil temperatures at 5 and 10 cm depths and the air temperature.

**FIGURE 6:**
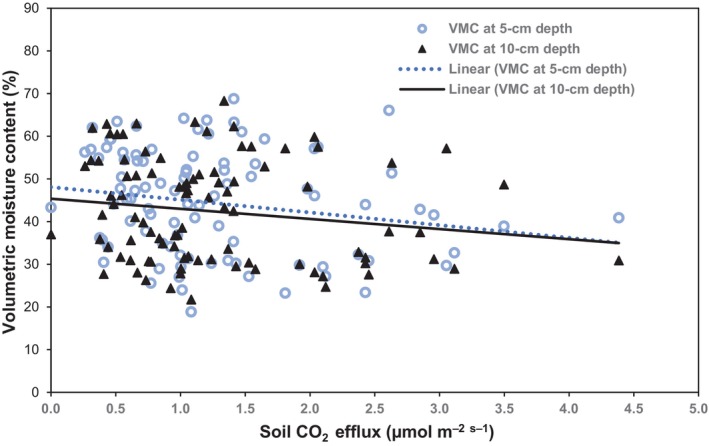
Soil respiration rates (μmol m^−2^ s^−1^) as a function of soil moisture (%) at 5 and 10 cm depths. The correlations show a weak negative relationship between R_s_ rates and soil moisture.

This study simultaneously measured the R_s_, soil temperature, volumetric soil moisture contents at 5 and 15 cm depths, and air temperature. The scatter plots demonstrated a good relationship between soil respiration, soil temperature, and air temperature but not with soil moisture (Figures 5 and 6). Pearson's correlation analysis revealed a moderately significant relationship between soil respiration and soil temperature at 5 cm depth (0.5936; *p* < .0001), 10 cm depth (0.5046; *p* < .0001), and air temperature (0.4684; *p* < .0001), suggesting that the R_s_ tends to increase with soil and air temperatures. However, soil respiration showed no relationship with soil moisture at 5 cm depth (−0.1983; *p* = .0527) and 10 cm depth (−0.1576; *p* = .1252), suggesting that it tends to decrease with increasing soil moisture contents.

Collecting R_s_ data directly in the field during the winter is challenging because of prohibitive weather conditions and difficulty accessing the research sites, particularly in remote and high‐elevation areas. Thus, installing temperature and moisture probes to monitor soil and air temperatures and soil moisture contents is a good alternative option to quantify R_s_ rates indirectly. Table 4 summarizes parameter estimates of soil temperature and soil moisture at 5 and 10 cm depths and air temperature. The criteria for selecting the most appropriate predictor variables for inclusion in the multiple linear regression model were based on the statistical analyses of *r* ^2^ values, Mallow's C_p_ statistics, Akaike's information criterion (AIC), and mean standard error (MSE). Variables with the highest *r* ^2^ but low values of Mallow's C_p_, AIC, and MSE are considered as best candidate variables for the multiple regression model. Results of the statistical analysis indicated that soil temperature at 5 cm depth, air temperature, and soil moisture content at 5 cm depth are appropriate independent variables to predict R_s_ rates, taking into account its relatively higher *r* ^2^ (.51) and low values of C_p_, AIC, and MSE. Including the soil temperature and moisture contents at 10 cm depths in the model does not significantly improve the precision of the model to estimate R_s_ rates (Table 5).

**TABLE 5:** Estimated parameters of line intercept of a regression line of a multiple regression model with soil temperature at 5 cm and 10 cm soil depths, air temperature, and soil moisture contents.

Variables	Parameter estimates	Standard error	*p*‐value
Intercept	1.6848	0.2337	<.0001
Soil temperature at 5 cm	0.1537	0.0418	.0004
Soil temperature at 10 cm	−0.0358	0.0474	.4512
Air temperature	0.0520	0.0142	.0004
Soil moisture contents at 5 cm (%)	−0.0182	0.0054	.0010
Soil moisture contents at 5 cm (%)	−0.0092	0.0056	.1061

## 4 DISCUSSION

### 4.1 Soil respiration rates

The postfire and undisturbed forests emitted CO_2_ from soils during winter in average amounts of 1.44 μmol m^−2^ s^−1^ for the snow undisturbed forest (SUF), 1.48 μmol m^−2^ s^−1^ for the snow‐exclusion‐undisturbed forest (SEUF), 1.02 μmol m^−2^ s^−1^ for the snow postfire forest (SPF), and 0.71 μmol m^−2^ s^−1^ for the snow‐exclusion postfire forest (SEPF) treatments (Table 3). The R_s_ rates of the postfire area compare well with some reported R_s_ rates during the winter season under different types of forest, with values ranging from 0.5 to 0.7‐μmol m^−2^ s^−1^ (e.g., Brooks et al., 1996; Coxson & Parkinson, 1987; Groffman et al., 2001; Liptzin et al., 2009; Nielsen et al., 2001; Pacaldo et al., 2014; Sommerfeld et al., 1993; Taylor & Jones, 1990; Tierney et al., 2001). In contrast, the undisturbed forest exhibited higher R_s_ rates than the reported R_s_ values, which could be explained by the warmer soil (2°C) during the winter (Figures 3 and 4). The warmer soil in the undisturbed forest during winter could be attributed to the presence of thick organic matter and snowpack. The soil organic matter has low thermal conductivity, and its relatively high heat capacity acts as an insulator for soil temperature that prevents significant heat losses (Decharme et al., 2016). The snowpack also insulates and protects the soil against direct contact with the ambient air and mitigates heat loss, thus keeping the soil warm and preventing soil freezing (Graham & Risk, 2018; Grogan & Jonasson, 2006; Ivanov et al., 2022; Monson et al., 2006), hence providing a favorable soil environment that allows uninterrupted microbial activities and respiration rates (Yang et al., 2021). Due to the protective layers of organic matter and snowpack, some authors explained that warmer soil promotes heterotrophic organism activities (Li et al., 2013), which continue to metabolize and respire even at −7°C soil temperatures (Flanagan & Bunnell, 1980).

In the case of snow‐exclusion treatment, the lack of forest litter and snowpack explained the low R_s_ rates in the postfire area due to freezing soil temperature. Although the warmer winter in Türkiye during this study resulted in intermittent snowfall and warmer soil, the soil temperature in the SEPF dropped at times to −2°C in the middle of winter (Figures 3 and 4). The freezing temperature probably explained the difference in R_s_ rates in the SEPF by about 30%–50% lower than the undisturbed forest and about 38% lower than the R_s_ of SPF (Table 3). These observations agree well with our prediction that the lack of snow and forest litter in the postfire sites could decrease the R_s_ rates because direct contact of the soil surface with ambient temperature results in cold soils and freezes the soil water, hence sealed soil pores and effectively reduced metabolic and respiration activities of soil microorganisms.

Furthermore, the lower R_s_ rates in the postfire area compared with the undisturbed forest could also be partly explained by the destructive effects of wildfires on soil organic matter, aboveground vegetation, roots, and soil organisms. Although some authors observed increased R_s_ rates in postfire areas during the growing season (e.g., Hu et al., 2021; Maljanen et al., 2006; Masyagina et al., 2016; Wang, Yao, et al., 2021) due to the release of nutrients (Ca, P, K, and Mg), except N, increased soil pH in the mineral soil layer due to ashes (Ernfors et al., 2010; Maljanen et al., 2006; Moilanen et al., 2012), improved litter quality (Stirling et al., 2019), and increased C/N ratio and electrical conductivity (EC) (Francos et al., 2019), the winter R_s_ rates showed a different trend, probably due to the combined inhibitory effects of destructive effects of wildfires and freezing temperature on R_s_ rates. Forest fires result in the destruction of the autotrophic and heterotrophic organisms in the active layer depths of the soil, alteration of the chemical and biological components (Masyagina et al., 2016), and reduction in the quantity of soluble C and microbial activities (Francos et al., 2019; Hobley et al., 2019). The forest fires also burned the light fraction organic carbon (LF‐OC) or labile carbon, the most active and dynamic C pool of particulate organic matter and microbial biomass, the primary sources of CO_2_ emissions through R_s_ (Post & Kwon, 2000). Furthermore, the wildfire also destroyed a large number of fine roots found close to the soil surface (Trumbore, 2006), hence reducing 10%–90% of the root's contribution to the total soil respiration rates (Cisneros‐Dozal et al., 2006). To the best of our knowledge, this finding provides the first evidence to show that a combination of forest fire effects and low temperature during the winter period results in a significant reduction in soil CO_2_ emissions, suggesting that lack of snow, as may occur due to climate change impacts, may not necessarily be harmful to the C stock deposits in postfire areas during winter time, but rather extenuates decomposition rates and, thus, the conservation of the soil organic carbon during this period.

In contrast, the uninterrupted soil organism activities during winter inevitably result in the mineralization of complex organic compounds to CO_2_ (Flanagan & Bunnell, 1980), hence losses of stored soil carbon. These losses could not be ignored or assumed to be insignificant in the calculation of the C budget because their quantity is quite large, which tends to increase with warmer soil during winter. In this study, the estimated total soil CO_2_ emissions during the entire winter season (January–March) were about 4.47 Mt CO_2_ ha^−1^ for the SEUF, 4.59 Mt CO_2_ ha^−1^ for the SUF, 3.16 Mt CO_2_ ha^−1^ for the SPF, and 2.20 Mt CO_2_ ha^−1^ for the SEPF. The soil organic carbon losses in the undisturbed forest ranged from 2.39 to 2.46 Mt OM ha^−1^, which is about 31% higher than the SPF (1.69 Mt OM ha^−1^) and 50% higher than the SEPF (1.18 Mt OM ha^−1^) treatments (Table 3). Some authors reported that winter R_s_ comprises about 30% of the total annual CO_2_ fluxes in sub‐alpine meadows (Liptzin et al., 2009) and about 5%–7% in the willow biomass crop plantation (Pacaldo, 2012). A complementary study showed that the contribution of winter R_s_ to the cumulative annual R_s_ in the study site was about 10% in the postfire and 13% in undisturbed forests (Pacaldo et al., 2024).

Inevitably, the results of this field‐based experiment increased our understanding of the feedback of R_s_ to compound effects of lack of snow and forest fire. However, these findings should be interpreted with caution due to uncertainties associated with responses of autotrophic and heterotrophic organisms to long‐term freezing and thawing cycles, high spatial variability of forest floor organic matter (Yanai et al., 2000), and soil nutrient limitations (Luo et al., 2022). Although our findings increase our understanding of the R_s_ feedback to the compound effects of lack of snow and forest fires, which better represent the entire ecosystem responses, these observations covered only a limited range of environmental conditions. Due to differences in processes and mechanisms across spatial and temporal scales, upscaling the results of these studies to regional and global scales poses a significant challenge, which could be alleviated by integration of findings of observational studies in controlled microcosms or laboratory‐based studies and field environments if the goal is to advance a detailed mechanistic and the underlying R_s_ processes at the soil–plant–organism level and increase prediction's accuracy of climate change impacts to R_s_ rates (Chen et al., 2023). Furthermore, this study also emphasized soil organic matter as a primary agent affecting temperature and R_s_ rates, which should also be interpreted with a caveat because the high spatial variability of soil organic matter stocks on the forest floor makes it difficult to distinguish whether the observed differences are more likely due to response to treatment or differences in the SOC stocks across the landscape (Yanai et al., 2000). Moreover, the short observation period in this study limits our ability to disentangle the R_s_ feedback to long‐term adjustments, adaptation, and acclimation of plant and soil organisms to a warmer climate, lack of snow, and cold winters (Chen et al., 2023).

### 4.2 Temperature changes with soil depths

Our second hypothesis was that a reduced snow cover or a lack thereof due to warmer winter results in colder soils and frequent freeze–thaw events. Results of this study showed that in the undisturbed natural forest, the winter soil temperatures of SUF and SEUF treatments, in 5 and 10 cm depths, remained above 2°C, except in the upper 5 cm depth during the mid‐winter period. In contrast, the SPF and SEPF plots in both soil depths in the postfire sites showed a decreasing soil temperature to zero at the mid‐winter and then rapidly increasing temperature during the last week of the winter season (Figures 3 and 4). The higher soil temperatures in the undisturbed plots could be explained by a thick layer of organic matter acting as a protective cover against cold air temperature at the soil surface and mitigating soil heat losses (Decharme et al., 2016). Microbial respiration, an exothermic reaction involving the generation and release of heat, also potentially contributed to the rise of soil temperature in the undisturbed forest during this freezing period (Campbell et al., 2005; Zimov et al., 1996). It is interesting to note that the lack of significant changes in the R_s_ rates in SEUF suggests that the soil microorganisms seem insensitive to slight differences in soil temperature, consistent with observations in some snow manipulation studies (Decker et al., 2003; Groffman et al., 2001; Tierney et al., 2001; Yang et al., 2021).

In contrast, the significantly lower temperature in the postfire plots could be attributed to a lack of forest litter that would insulate the soil surface against direct contact with freezing air temperature and snow. The direct exposure of the soil surface to ambient temperature resulted in the freezing temperature (0°C) at the soil surface (5 cm depth) and −2°C at the subsurface (Figures 3 and 4). Unexpectedly, the SEPF plots exhibited a negative soil temperature at the height of the winter season at a depth of 10 cm, which was lower than in the upper 5 cm depth. This delay in reaction to the warmer air temperature may be related to the hysteresis effect or lag time in the change of temperature in the deeper soil depth, considering that the negative temperature was recorded during snow thawing due to warm air temperature. Chu et al. (2023) observed that the lag time between R_s_ and soil temperature ranged from 1 to 8 h.

Notably, the soil temperature in the 10 cm depth of the SEPF reached below −2°C in the mid‐winter season. The observed R_s_ rates in the SEPF, even at freezing temperature, suggest the ability of soil microorganisms to adjust to freezing soil temperature and be less sensitive to short‐term soil temperature variability (Yang et al., 2021). Some authors explained that the CO_2_ emissions continue during the winter period despite a freezing temperature because of the ability of soil organisms to adjust and adapt their metabolic processes even at −7°C soil temperatures (Brooks et al., 1996; Coxson & Parkinson, 1987; Flanagan & Bunnell, 1980; Graham et al., 2018).

The preceding observations suggest that cold soil in a warm winter could potentially negatively impact the growth of young forests, particularly in the rehabilitation of postfire areas using a natural regeneration approach. A warm winter with cold soils drives frequent freeze–thaw cycles, inevitably resulting in root injury and mortality of newly grown wildlings. Decker et al. (2003) reported a similar observation in a snow study in a deciduous forest where snow‐free soils exhibited colder soils than the control. This condition could result in root mortality, nutrient loss, ecosystem dynamic alteration, and decreased productivity in some trees. The possible consequences could be more serious in a severe freeze that directly causes root and microbial mortality (Groffman et al., 2001; Nielsen et al., 2001).

### 4.3 Relationship of soil respiration with temperature and soil moisture

Temperature and soil moisture are complementary regulatory factors influencing R_s_ rates (Cui et al., 2020; Dinca et al., 2018). In this study, the winter R_s_ showed moderate correlations with soil temperature at 5 cm depth (*r* = .5936; *p* < .0001) and 10 cm depth (*r* = .5046; *p* < .0001) and air temperature (*r* = .4684; *p* < .0001) and a slight negative correlation with soil moisture at 5 cm depth (*r* = −.1983; *p* = .0527) and 10 cm depth (*r* = −.1576; *p* = .1252) (Figures 5 and 6). The positive correlation between R_s_ and temperature indicates the tendency of R_s_ rates to increase with increasing soil and air temperatures. Some studies demonstrated that the soil temperature exerts a dominant influence on R_s_ rates during the growing season when soil moisture is not a limiting factor of plant growth (Fei et al., 2015; Janssens et al., 2001) because of the strong influence of temperature on autotrophic and heterotrophic activities (Raich & Schlesinger, 1992). The air temperature also showed a moderate positive relationship with winter R_s_, suggesting that it approximates R_s_ well. This finding could address the limitations of continuously monitoring soil temperature and moisture on large scales and over long periods. Given the abundant data on air temperature in many meteorological stations, it becomes more convenient to model the trajectory of R_s_ rates using air temperature data, particularly in light of the projected increase in air temperature with global warming (Amthor, 2000; Lewis, 2006).

Findings on the influence of soil moisture on R_s_ rates are not consistent, with some authors reporting a positive relationship (Fei et al., 2015; Raich & Schlesinger, 1992; Wood et al., 2013), a negative relationship (Adachi et al., 2005; Yanni et al., 2020), and no significant relationship (Borken et al., 2006; Bréchet et al., 2009). In this study, the lack of any explanatory effects of soil moisture on R_s_ rates (Figure 6) could be related to the concealment of soil water effects on R_s_ when water is abundant and not a limiting factor (Yanni et al., 2020). Also, when the soil temperature dropped below 0°C, the frozen soil inhibited the diffusion of oxygen and CO_2_, which prevented the detection of any changes in R_s_ rates above the surface of the frozen soil layers.

In the prediction of the trajectory of R_s_ rates during the winter season, the soil temperature at 5 cm depth, air temperature, and soil moisture content at 5 cm depth could be used as independent variables to predict R_s_ rates, taking into account its relatively higher *r* ^2^ (.51) and low values of C_p_, AIC, and MSE (Table 6). Thus, a linear regression model to predict R_s_ rates could be *Y* = 1.6848 + 0.537 (SoilT_5‐cm_) + 0.0520 (AirT) − 0.0182 (VMC_5‐cm_) (Table 5). Although the utilization of air temperature as the only variable to predict R_s_ rates is more convenient due to an abundance of data and availability in meteorological stations, the analysis revealed that it only explains about 21% of the R_s_ variability and demonstrated a higher C_p_, AIC, and MSE, suggesting that it is not a stand‐alone variable for the prediction of R_s_ rates. However, this should be interpreted with caution because other factors may also exert an influence on R_s_ rates (Chen et al., 2016; Wang, Yao, et al., 2021). Furthermore, high‐*r* ^2^ values do not necessarily mean good causal relationships.

**TABLE 6:** Estimated *r* ^2^ values, Mallows' C_p_ statistics, Akaike's information criterion (AIC), and mean standard error (MSE).

Variables in the model	*r* ^2^	C_p_	AIC	MSE	Independent variables
1	.35	32.49	−107.97	0.32	ST5
1	.25	51.27	−94.51	0.37	ST15
1	.21	58.04	−90.07	0.38	AirT
1	.04	92.66	−70.15	0.47	VMC5
1	.02	95.45	−68.71	0.48	VMC10
2	.46	13.88	−123.37	0.27	ST5 and VMC5
3	.51	5.49	−131.47	0.24	ST5, AirT, and VMC5
4	.53	4.57	−132.53	0.24	ST5, AirT, VMC5, and VMC10
5	.53	6.00	−131.14	0.24	ST5, ST10, AirT, VMC5, and VMC10

Abbreviations: AirT, ambient temperature; ST5, soil temperature at 5 cm depth; ST10, soil temperature at 10 cm depth; VMC5, volumetric moisture content at 5 cm depth; VMC10, volumetric moisture content at 15 cm depth.

Contrarily, forest fires exert little influence on the soil temperature and soil moisture during the winter period. Although some studies demonstrated that wildfires drive warmer temperatures and drier soil conditions (Flanagan & Bunnell, 1980), the current study showed that the postfire area had a significantly lower temperature than the undisturbed forest (control). Also, the soil temperature in the SEPF dropped to −2°C during the mid‐winter season, suggesting little effect of forest fire in regulating and controlling the soil temperature and moisture during winter. We predicted that a forest fire would strongly influence moisture contents due to its direct impacts on soil hydrologic conductivity and increased hydrophobicity due to the ash deposits. Wittenberg et al. (2020) observed that in a high‐intensity forest fire, the hydraulic conductivity (K) of the black ash, white ash, and disturbed mixed ash reduced the soil infiltration rates, resulting in a drier subsurface soil layer. In this study, however, no significant differences in the soil moisture contents were detected in the upper 5 cm (ash layer) and 10 cm depths (Figure 6). One possible explanation is the effects of the natural recovery of the site, taking into account that 4 years had passed since the forest fire consumed the site.

Moreover, grasses, shrubs, and tree wildlings have been invading the postfire sites during this study. Although forest fires cause the reduction in the organic matter layer and hydrophobicity of soil, it is possible that the magnitude of hydrophobicity effects on soil water infiltration rates eventually decreases until such time that the presence of ashes on the soil surface no longer affects soil water infiltration rates. This observation agrees well with the findings of Kong et al. (2019), in which forest fires had no significant impacts on soil properties and postfire sites recovered rapidly to their state during the pre‐fire period except for soil organic matter and microbes.

## 5 CONCLUSION

The complementary effects of lack of snow and forest fire during winter caused a significant decrease in R_s_ rates and soil temperature. Evidence of this study revealed that the snow‐exclusion postfire forest (SEPF) exhibited the significantly lowest soil temperature (−2°C) and R_s_ rates (0.71 μmol m^−2^ s^−1^). The lack of snow alone is insufficient to drive significant changes in the R_s_ in the undisturbed forests, as exhibited by non‐significant differences in R_s_ rates between snow undisturbed forest (SUF) or control and snow‐exclusion‐undisturbed forest (SEUF) plots. The undisturbed forest also showed high R_s_ rates, ranging from 1.44 to 1.48 μmol m^−2^ s^−1^, which is about twice as high as usually observed R_s_ rates during the winter period (e.g., Groffman et al., 2001; Liptzin et al., 2009; Pacaldo et al., 2014; Sommerfeld et al., 1993; Tierney et al., 2001), suggesting that a warmer winter results in elevated R_s_ in the undisturbed natural forests. The snow is an essential regulator in alleviating adverse impacts of freezing air temperature, consistent with our initial prediction that the snow acts as an effective soil insulator in forest fire sites and undisturbed forests and, thus, the lack of snow results in cold soils, decreased R_s_ rates, and potential damage to roots and mortality of soil organisms.

Lack of snow and freezing air temperatures in warmer winters positively and negatively impact soil C stocks. In the postfire areas, the direct exposure of the mineral soil to the freezing ambient temperature results in a freezing soil temperature and a significant decrease in R_s_ rates, thus potentially conserving the soil organic carbon stocks and reducing the CO_2_ contribution into the atmosphere. In contrast, the undisturbed forest exhibited higher R_s_ rates, with or without snow cover, suggesting that a warmer winter could accelerate soil organic carbon losses and, thus, positive feedback to climate change.

The soil and air temperatures moderately correlated with R_s_ rates in postfire and undisturbed forest sites. However, the best predictor variables of the R_s_ are the soil temperature at 5 cm depth, air temperature, and soil moisture at 5 cm depth.

These findings also highlight the importance of incorporating the winter R_s_ in estimating the forest carbon balance, which regrettably is not usually accounted for in many forest carbon budget estimates due to limited data, challenges associated with the R_s_ measurements in cold and wet harsh conditions (Graham & Risk, 2018), and the common assumption that the winter R_s_ constitutes only a tiny fraction of the total annual C budgets. This study proves that the winter R_s_ (January–March) rates are high in the black pine forest, ranging from 4.45 to 4.59 Mt CO_2_ ha^−1^ in the undisturbed forest and 2.20 to 3.17 Mt CO_2_ ha^−1^ in the postfire areas. These contributions must be accounted for in accurate C budgets accounting in the black pine forest ecosystem. A complementary study found that the winter R_s_ of undisturbed forests contributed about 13%, while the postfire was about 10% to the total annual cumulative R_s_ rates in the black pine forest ecosystem (Pacaldo et al., 2024).

While our snow‐exclusion treatments, both in the postfire site and undisturbed forest, showed a significant decrease in R_s_ rates and the soil temperature, this should be interpreted with caution due to uncertainties on the detailed mechanisms of autotrophic and heterotrophic R_s_ feedback to short freezing and thawing cycles (Luo et al., 2022) and high spatial variability of forest floor organic matter (Yanai et al., 2000). Furthermore, a more accurate prediction on the trajectory of the R_s_ feedbacks to warmer and snow‐free winter and upscaling this observation to regional and global scales need a holistic understanding of the underlying mechanisms of the processes and functional relationship of soil–plant–microorganisms at different levels, which requires the integration of findings in both field‐based and laboratory or microcosm studies (Chen et al., 2023). Understanding the details of these mechanisms has important implications not only for predicting the trajectory of R_s_‐climate change feedback but also for the management of newly growing wildlings in forest fire areas whose survival could be affected by short‐term alternate freezing and thawing cycles (Decker et al., 2003; Groffman et al., 2001; Nielsen et al., 2001) and crafting the management and rehabilitation plan for forest fire areas. Further studies need to be conducted to determine the long‐term impacts of lack of snow and frequent freeze–thaw cycles on the health and survival of black pine wildlings, recovery of forest fire areas, and adaptation and acclimation of plant and microbial organisms as affected by compound effects of lack of snow and forest fires.

## AUTHOR CONTRIBUTIONS

**Renato S. Pacaldo:** Conceptualization (lead); data curation (lead); formal analysis (lead); funding acquisition (lead); investigation (lead); methodology (lead); project administration (lead); resources (lead); supervision (lead); validation (lead); visualization (lead); writing – original draft (lead); writing – review and editing (lead). **Mirac Aydin:** Conceptualization (supporting); data curation (supporting); formal analysis (supporting); funding acquisition (equal); investigation (supporting); methodology (supporting); project administration (equal); resources (supporting); supervision (supporting); validation (supporting); writing – original draft (supporting); writing – review and editing (supporting). **Randell Keith Amarille:** Conceptualization (supporting); data curation (supporting); formal analysis (supporting); investigation (supporting); methodology (supporting); resources (supporting); validation (supporting); writing – original draft (supporting); writing – review and editing (supporting).

## FUNDING INFORMATION

This study was supported by the Türkiye Bilimsel ve Teknolojik Araştırma Kurumu (TÜBİTAK), the Science and Fellowship Grant No. 121C066, under the CoCirculation2 with funding from the European Union's Horizon 2020 research and innovation program under the Marie Sklodowska‐Curie Grant Agreement No. 801509.

## CONFLICT OF INTEREST STATEMENT

The authors declare no conflicts of interest.

## Supporting information


Appendix S1

## Data Availability

All data used in this work are available in the Appendix S1 of this article.
