# Supplementary material for: Soil respiration and controls in warmer winter: A snow manipulation study in postfire and undisturbed black pine forests
Source: Ecol Evol. 2024 Mar 6;14(3):e11075. doi: 10.1002/ece3.11075 (PMC10917581; doi:10.1002/ece3.11075)
Supplement: Supplementary file 1 — Appendix S1 [file ECE3-14-e11075-s001.docx]

**APPENDIX**

Data of soil respiration rates, soil temperature at 5 and 10 cm depths, air temperature, and soil moisture contents from January to March 2023 (winter months)

| TREATMENT | TIME | Plot | Flux (µmol m^-2^ s^-1^) | SoilTemp 5-cm (°C) | [SoilTemp 10-cm (°C)](mailto:SoilT@10cm) | AirTemp (°C) | [VMC at 5-cm Depth (%)](mailto:SoilM@5cm) | [VMC at 10-cm Depth (%)](mailto:SoilM@10cm) |
| --- | --- | --- | --- | --- | --- | --- | --- | --- |
| SPF | 1 | 1 | 1.34 | 9.05 | 9.05 | 12.67 | 53.68 | 68.28 |
| SPF | 1 | 2 | 1.20 | 5.57 | 5.57 | 15.33 | 63.75 | 61.16 |
| SPF | 1 | 3 | 2.96 | 9.05 | 9.05 | 12.50 | 41.54 | 31.19 |
| SPF | 1 | 4 | 1.47 | 5.57 | 5.57 | 15.11 | 61.03 | 57.67 |
| SPF | 2 | 1 | 0.59 | 3.07 | 3.74 | 1.47 | 45.12 | 50.64 |
| SPF | 2 | 2 | 0.65 | 1.01 | 1.36 | 2.13 | 47.27 | 41.03 |
| SPF | 2 | 3 | 1.10 | 3.61 | 3.25 | 1.63 | 55.27 | 49.96 |
| SPF | 2 | 4 | 0.67 | 1.20 | 0.75 | 2.12 | 54.22 | 28.05 |
| SPF | 3 | 1 | 0.98 | 2.62 | 3.79 | 0.66 | 36.85 | 36.85 |
| SPF | 3 | 2 | 1.14 | 0.14 | 0.66 | 1.75 | 43.82 | 51.04 |
| SPF | 3 | 3 | 0.86 | 2.62 | 3.79 | 0.41 | 34.88 | 34.88 |
| SPF | 3 | 4 | 0.92 | 0.02 | -0.12 | 1.36 | 34.12 | 24.41 |
| SPF | 4 | 1 | 0.78 | 1.11 | 2.17 | -2.40 | 56.90 | 51.35 |
| SPF | 4 | 2 | 0.99 | 0.63 | 1.45 | -1.20 | 26.98 | 48.12 |
| SPF | 4 | 3 | 0.37 | 2.00 | 1.90 | -2.35 | 54.88 | 54.25 |
| SPF | 4 | 4 | 0.73 | 0.47 | 1.00 | -1.52 | 37.76 | 26.27 |
| SPF | 5 | 1 | 1.58 | 5.25 | 4.57 | 6.49 | 53.50 | 28.84 |
| SPF | 5 | 2 | 0.57 | 2.96 | 3.34 | 0.57 | 54.46 | 54.52 |
| SPF | 5 | 3 | 0.89 | 3.70 | 3.55 | 2.43 | 57.40 | 47.94 |
| SPF | 5 | 4 | 0.51 | 2.88 | 2.75 | 0.24 | 63.47 | 60.45 |
| SPF | 6 | 1 | 2.61 | 7.30 | 5.10 | 8.59 | 66.04 | 37.69 |
| SPF | 6 | 2 | 0.85 | 4.35 | 4.37 | 7.45 | 48.97 | 54.89 |
| SPF | 6 | 3 | 1.03 | 6.29 | 8.02 | 9.04 | 64.19 | 48.16 |
| SPF | 6 | 4 | 1.41 | 4.21 | 4.91 | 7.74 | 68.78 | 62.33 |
| SEPF | 1 | 1 | 1.26 | 3.01 | 4.87 | 12.42 | 45.97 | 51.61 |
| SEPF | 1 | 2 | 0.73 | 3.01 | 4.87 | 14.65 | 48.01 | 56.43 |
| SEPF | 1 | 3 | 1.81 | 3.01 | 4.87 | 12.23 | 23.24 | 57.14 |
| SEPF | 1 | 4 | 0.71 | 3.01 | 4.87 | 14.25 | 54.11 | 39.79 |
| SEPF | 2 | 1 | 0.00 | 3.59 | 3.71 | 1.83 | 43.32 | 37.00 |
| SEPF | 2 | 2 | 0.26 | 2.32 | 2.45 | 2.25 | 56.24 | 52.99 |
| SEPF | 2 | 3 | 0.61 | 3.84 | 3.77 | 1.85 | 40.11 | 30.92 |
| SEPF | 2 | 4 | 0.55 | 2.13 | 2.24 | 2.53 | 50.41 | 46.20 |
| SEPF | 3 | 1 | 0.48 | 3.97 | 4.56 | 0.43 | 44.10 | 44.10 |
| SEPF | 3 | 2 | 0.40 | 1.83 | 2.15 | 1.80 | 35.72 | 41.60 |
| SEPF | 3 | 3 | 0.44 | 3.97 | 4.56 | 0.72 | 34.04 | 34.04 |
| SEPF | 3 | 4 | 0.62 | 1.49 | 1.76 | 2.55 | 45.31 | 35.62 |
| SEPF | 4 | 1 | 0.38 | 0.97 | 1.70 | -2.21 | 36.20 | 35.88 |
| SEPF | 4 | 2 | 0.77 | -0.18 | 0.31 | -1.55 | 25.58 | 30.60 |
| SEPF | 4 | 3 | 0.75 | 0.49 | -2.53 | -2.41 | 42.99 | 30.68 |
| SEPF | 4 | 4 | 2.10 | -0.19 | 0.22 | -1.57 | 29.39 | 27.17 |
| SEPF | 5 | 1 | 1.14 | 4.26 | 5.10 | 6.73 | 61.62 | 30.93 |
| SEPF | 5 | 2 | 0.66 | 3.21 | 3.46 | 0.66 | 55.62 | 50.81 |
| SEPF | 5 | 3 | 0.70 | 3.55 | 4.08 | 2.88 | 58.05 | 45.37 |
| SEPF | 5 | 4 | 0.31 | 3.19 | 3.67 | 1.25 | 56.90 | 54.38 |
| SEPF | 6 | 1 | 1.34 | 6.72 | 8.19 | 9.24 | 52.02 | 43.28 |
| SEPF | 6 | 2 | 0.46 | 4.74 | 3.99 | 8.10 | 59.33 | 46.05 |
| SEPF | 6 | 3 | 1.05 | 4.91 | 6.47 | 9.39 | 52.15 | 49.01 |
| SEPF | 6 | 4 | 1.04 | 5.77 | 4.37 | 8.33 | 51.52 | 46.65 |
| SUF | 1 | 1 | 2.63 | 12.90 | 7.81 | 12.80 | 51.37 | 53.74 |
| SUF | 1 | 2 | 1.11 | 4.36 | 7.81 | 11.37 | 40.87 | 63.33 |
| SUF | 1 | 3 | 2.85 | 12.90 | 6.46 | 13.25 | 42.85 | 37.48 |
| SUF | 1 | 4 | 3.11 | 6.50 | 6.46 | 11.19 | 32.68 | 28.94 |
| SUF | 2 | 1 | 1.36 | 4.63 | 5.33 | 1.42 | 48.82 | 47.02 |
| SUF | 2 | 2 | 1.41 | 2.78 | 2.66 | 3.88 | 35.31 | 42.45 |
| SUF | 2 | 3 | 2.37 | 3.86 | 3.20 | 1.51 | 32.27 | 32.88 |
| SUF | 2 | 4 | 0.43 | 2.45 | 2.90 | 3.80 | 57.36 | 62.88 |
| SUF | 3 | 1 | 1.00 | 4.45 | 5.50 | 1.20 | 32.11 | 27.83 |
| SUF | 3 | 2 | 1.43 | 1.73 | 2.77 | 1.52 | 30.22 | 29.52 |
| SUF | 3 | 3 | 1.92 | 4.14 | 6.71 | 0.91 | 29.80 | 30.03 |
| SUF | 3 | 4 | 0.56 | 2.11 | 2.58 | 1.46 | 56.17 | 60.49 |
| SUF | 4 | 1 | 1.08 | 2.68 | 4.08 | -1.17 | 18.85 | 21.74 |
| SUF | 4 | 2 | 1.06 | 1.26 | 1.57 | -0.79 | 31.34 | 31.88 |
| SUF | 4 | 3 | 1.08 | 2.68 | 4.08 | -1.35 | 18.85 | 21.74 |
| SUF | 4 | 4 | 0.54 | 1.51 | 2.10 | -0.66 | 47.75 | 31.70 |
| SUF | 5 | 1 | 0.32 | 4.26 | 4.83 | 4.70 | 61.99 | 61.94 |
| SUF | 5 | 2 | 1.65 | 2.32 | 3.73 | 8.34 | 59.34 | 52.90 |
| SUF | 5 | 3 | 2.03 | 4.97 | 5.55 | 4.76 | 46.05 | 28.11 |
| SUF | 5 | 4 | 0.46 | 2.82 | 3.78 | 6.91 | 59.24 | 60.65 |
| SUF | 6 | 1 | 2.06 | 6.18 | 5.12 | 8.37 | 57.49 | 57.50 |
| SUF | 6 | 2 | 2.03 | 3.97 | 3.70 | 12.36 | 57.10 | 59.84 |
| SUF | 6 | 3 | 2.43 | 5.69 | 6.47 | 8.73 | 43.97 | 31.56 |
| SUF | 6 | 4 | 0.66 | 3.55 | 3.92 | 13.01 | 62.36 | 62.97 |
| SEUF | 1 | 1 | 4.38 | 5.44 | 5.29 | 14.42 | 40.88 | 30.89 |
| SEUF | 1 | 2 | 1.55 | 5.44 | 5.29 | 11.05 | 50.54 | 57.58 |
| SEUF | 1 | 3 | 3.50 | 5.44 | 4.26 | 14.52 | 38.92 | 48.67 |
| SEUF | 1 | 4 | 1.98 | 5.44 | 4.26 | 10.83 | 47.67 | 48.19 |
| SEUF | 2 | 1 | 2.45 | 5.31 | 5.53 | 1.61 | 30.82 | 27.59 |
| SEUF | 2 | 2 | 0.96 | 2.91 | 3.24 | 3.42 | 47.21 | 36.75 |
| SEUF | 2 | 3 | 1.53 | 5.52 | 5.79 | 1.88 | 27.15 | 30.38 |
| SEUF | 2 | 4 | 1.30 | 2.49 | 2.89 | 3.09 | 38.98 | 49.16 |
| SEUF | 3 | 1 | 2.12 | 5.34 | 6.10 | 0.88 | 27.18 | 24.69 |
| SEUF | 3 | 2 | 0.77 | 2.84 | 3.10 | 0.88 | 41.72 | 37.55 |
| SEUF | 3 | 3 | 1.37 | 4.67 | 5.80 | 1.12 | 30.88 | 33.58 |
| SEUF | 3 | 4 | 1.06 | 2.13 | 2.59 | 0.73 | 44.08 | 47.31 |
| SEUF | 4 | 1 | 1.00 | 2.24 | 2.41 | -1.37 | 29.45 | 28.97 |
| SEUF | 4 | 2 | 0.41 | 0.35 | 0.52 | -0.69 | 30.45 | 27.70 |
| SEUF | 4 | 3 | 1.00 | 2.24 | 2.41 | -0.85 | 29.45 | 28.97 |
| SEUF | 4 | 4 | 1.01 | 0.11 | 0.61 | -0.71 | 23.97 | 38.52 |
| SEUF | 5 | 1 | 2.43 | 5.67 | 5.55 | 5.13 | 23.38 | 30.22 |
| SEUF | 5 | 2 | 0.95 | 3.32 | 3.95 | 5.91 | 39.74 | 34.22 |
| SEUF | 5 | 3 | 1.24 | 5.71 | 5.80 | 5.84 | 30.22 | 31.15 |
| SEUF | 5 | 4 | 1.22 | 3.14 | 4.05 | 6.19 | 60.53 | 45.61 |
| SEUF | 6 | 1 | 3.05 | 5.98 | 5.31 | 9.21 | 29.70 | 57.16 |
| SEUF | 6 | 2 | 0.84 | 4.69 | 4.34 | 13.04 | 28.94 | 36.08 |
| SEUF | 6 | 3 | 1.03 | 5.60 | 5.66 | 9.50 | 50.23 | 31.46 |
| SEUF | 6 | 4 | 1.41 | 3.77 | 4.38 | 11.85 | 63.26 | 49.40 |

**SPF** – Snow (i.e., exposed to natural snowfall) in PostFire Plots (i.e., forested area gutted by crown fire with all trees burned and removed)

**SEPF** – Snow Exclusion (i.e. covered with snow shelter) in PostFire Plots

**SUF** - Snow (i.e exposed to natural snowfall) in Undisturbed Forest Plots (forest bordering the forest fire area, which was not affected by forest fire during)

**SEUF** – Snow Exclusion in Undisturbed Forest

**Time of Measurements:**

1 and 2: January

3 and 4: February

5 and 6: March
